# Co-modulation of TNFR1 and TNFR2 in an animal model of multiple sclerosis

**DOI:** 10.1186/s12974-023-02784-z

**Published:** 2023-04-30

**Authors:** Timon Fiedler, Richard Fairless, Kira Pichi, Roman Fischer, Fabian Richter, Roland E. Kontermann, Klaus Pfizenmaier, Ricarda Diem, Sarah K. Williams

**Affiliations:** 1 Department of Neurology, University Clinic Heidelberg, University of Heidelberg, Otto-Mayerhof-Zentrum (OMZ), Im Neuenheimer Feld 350, 69120 Heidelberg, Germany; 2 Clinical Cooperation Unit (CCU) Neurooncology, German Cancer Consortium (DKTK), German Cancer Research Center (DKFZ), 69120 Heidelberg, Germany; 3 Institute of Cell Biology and Immunology, University of Stuttgart, Allmandring 31, 70569 Stuttgart, Germany; 4 Present Address: BioNtech SE, An der Goldgrube 12, 55131 Mainz, Germany; 5 Present Address: Immatics Biotechnologies GmbH, Paul-Ehrlich-Str. 15, 72076 Tübingen, Germany

**Keywords:** EAE, TNFR1, TNFR2, Neuroinflammation, Neuroprotection

## Abstract

**Background:**

Tumour necrosis factor (TNF) is a pleiotropic cytokine and master regulator of the immune system. It acts through two receptors resulting in often opposing biological effects, which may explain the lack of therapeutic potential obtained so far in multiple sclerosis (MS) with non-receptor-specific anti-TNF therapeutics. Under neuroinflammatory conditions, such as MS, TNF receptor-1 (TNFR1) is believed to mediate the pro-inflammatory activities associated with TNF, whereas TNF receptor-2 (TNFR2) may instead induce anti-inflammatory effects as well as promote remyelination and neuroprotection. In this study, we have investigated the therapeutic potential of blocking TNFR1 whilst simultaneously stimulating TNFR2 in a mouse model of MS.

**Methods:**

Experimental autoimmune encephalomyelitis (EAE) was induced with myelin oligodendrocyte glycoprotein (MOG_35-55_) in humanized TNFR1 knock-in mice. These were treated with a human-specific TNFR1-selective antagonistic antibody (H398) and a mouse-specific TNFR2 agonist (EHD2-sc-mTNF_R2_), both in combination and individually. Histopathological analysis of spinal cords was performed to investigate demyelination and inflammatory infiltration, as well as axonal and neuronal degeneration. Retinas were examined for any protective effects on retinal ganglion cell (RGC) degeneration and neuroprotective signalling pathways analysed by Western blotting.

**Results:**

TNFR modulation successfully ameliorated symptoms of EAE and reduced demyelination, inflammatory infiltration and axonal degeneration. Furthermore, the combinatorial approach of blocking TNFR1 and stimulating TNFR2 signalling increased RGC survival and promoted the phosphorylation of Akt and NF-κB, both known to mediate neuroprotection.

**Conclusion:**

These results further support the potential of regulating the balance of TNFR signalling, through the co-modulation of TNFR1 and TNFR2 activity, as a novel therapeutic approach in treating inflammatory demyelinating disease.

**Supplementary Information:**

The online version contains supplementary material available at 10.1186/s12974-023-02784-z.

## Introduction

Tumour necrosis factor α (TNF) is a pleotropic cytokine which is a considered to be a master regulator of the inflammatory response. It is primarily produced by macrophages and lymphocytes and is implicated in the pathogenesis and progression of a number of autoimmune conditions, including rheumatoid arthritis and inflammatory bowel disease [1] as well as multiple sclerosis (MS), where it is upregulated in MS brains [2].

TNF signals through two receptors, TNF receptor-1 (TNFR, p55/p60) and TNF receptor-2 (TNFR2, p75/p80), and is synthesized as a membrane-bound protein (tmTNF) before subsequent cleavage by TNF-α-converting enzyme (TACE)/ADAM17 to produce soluble TNF (sTNF). tmTNF can activate both TNFR1 and TNFR2, whereas sTNF predominantly activates TNFR1. In recent years it has become evident that very different and often opposing biological functions can be elicited by TNF pursuant to its activation of either TNFR1 or TNFR2 [3]. In addition to promoting pro-inflammatory responses, the almost ubiquitously expressed TNFR1 contains a death domain in its cytoplasmic region, and can mediate apoptosis and necroptosis. However, TNF signalling via TNFR2, which has a much more restricted and regulated pattern of expression, can lead to anti-inflammatory effects, as well as neuroprotection and remyelination [4].

The activity of these opposing receptors may in part explain the failure of an anti-TNF therapeutic, lenercept, to treat MS in a phase II clinical trial, instead resulting in aggravated demyelination [5]. Similarly, anti-TNF therapeutics have been associated with severe side effects, such as exacerbated inflammation, opportunistic infections, reactivation of tuberculosis and the development of autoimmune disease [4].

Therefore, to avoid inhibition of potentially protective TNFR2 signalling, therapeutics have since been developed to selectively target TNFR1, successfully reducing inflammation in several animal disease models [6–8]. For example, we and others have previously demonstrated the efficacy of anti-TNFR1 antibody antagonism in experimental autoimmune encephalomyelitis (EAE), an animal model of MS [9–13].

Given the divergent biological effects of the two TNFRs, one logical next step is to simultaneously block TNFR1 whilst also promoting TNFR2 signalling through the application of a selective TNFR2 agonist [4]. Since TNFR2 activation has been directly linked to neuroprotection, for example protecting cultured neurons exposed to H_2_O_2_ [14] or *N*-methyl-d-aspartate (NMDA) [15] from cell death, it could be hypothesized that the dual targeting of TNFR1 and TNFR2 may achieve greater efficacy in terms of both immunosuppression and neuroprotection under neuroinflammatory conditions than targeting the receptors in isolation.

Here we report on the therapeutic potential of dual TNFR1-selective antagonism and TNFR2 agonism using the human TNFR1-selective antagonistic antibody H398 [16] and the mouse TNFR2-specific fusion protein EHD2-sc-mTNF_R2_ [17] in humanized TNFR1 mice induced with an animal model of multiple sclerosis.

## Materials and methods

### Animals

Humanized TNFR1 knock-in (hu/m TNFR1-ki) mice were generated by Ozgene Pty Ltd as previously described [15] and were kept under environmentally controlled conditions in the absence of pathogens. All animal work was performed in accordance with European and German animal protection law with approval from the ‘Regierungspräsidium’ in Karlsruhe, Germany.

### Induction and evaluation of EAE and treatment of mice

EAE was induced as previously described [11, 12, 18]. Only female mice were included in this study in order to generate a homogenous population for comparison purposes (avoiding potentially confounding effects of sex hormones on the underlying disease pathophysiology [19, 20] and to potentially reflect the higher incidence of MS in women than men [21]). Briefly, mice 8–10 weeks of age were immunized subcutaneously with 300 µg MOG_35-55_ in complete Freund’s adjuvant (Sigma-Aldrich, St. Louis, MO, USA) containing 10 mg/ml heat-inactivated *Mycobacterium tuberculosis* H37RA (Difco microbiology, Lawrence, KS, USA) and received intra-peritoneal injection of 300 ng pertussis toxin (Enzo Life Science, Lörrach, Germany) immediately afterwards and again 2 days later. Mice were weighed and scored on a daily basis using a scale ranging from 0 to 5: 0, no clinical disease; 0.5, distal tail paresis; 1.0, complete tail paralysis; 1.5, tail paresis and mildly impaired righting reflex; 2.0, gait ataxia and severely reduced righting reflex; 2.5, bilateral severe hind limb paresis; 3.0, complete bilateral hind limb paralysis; 3.5, complete bilateral hind limb paralysis and forelimb paresis; 4, fore limb paralysis; 5, moribund state or death. In order to reduce development of anti-drug antibody responses in EAE mice, we applied TNFR selective ant/-agonist of mouse origin. Accordingly, mice received intra-peritoneal injections of either 20 mg/kg of the human TNFR1-selective antagonistic antibody H398 [16], 10 mg/kg of the murine homolog of a TNFR2-selective agonist EHD2-sc-mTNF_R2_ [17] or a combination of both, in a total volume of 300 µl, on days 1, 4, 8, 12 and 16 of manifest EAE. Control mice received intra-peritoneal injections of 300 µl phosphate-buffered saline (PBS). EAE experiments were performed on 5 separate occasions and data pooled.

### Spinal cord histopathology

Mice received an overdose of ketamine/xylazine and were transcardially perfused with 4% paraformaldehyde (PFA) in PBS. Spinal cords were dissected, processed for paraffin-embedding and 0.5 µm transverse sections were cut, with 10 sections per stain taken at regular intervals to cover the whole spinal cord. Luxol fast blue (LFB) staining was performed in order to assess demyelination, as previously described [12]. For immunohistochemistry, antigen retrieval was performed by incubating tissue sections in heated (~ 80 °C) 0.2% citrate buffer (pH 6.0) for 15 min, before being left to cool. Antibodies against Mac-3 (1:200, BD Biosciences, San Jose, CA, USA; Cat# 550292, RRID:AB_393587), CD3 (1:150, Agilent, Santa Clara CA, USA; Cat# A0452, RRID:AB_2335677), and FoxP3 (1:100, Thermo Fisher Scientific, Waltham, MA, USA; Cat# 14-5773-80, RRID:AB_467575), were used to detect activated microglia/macrophages, T cells, and regulatory T cells, respectively. Further antibodies were used to detect differentiated/myelinating oligodendrocyte cell bodies (anti-adenomatous polyposis coli (APC) (Ab-7) clone CC1, Merck Millipore, Darmstadt, Germany; Cat# OP80, RRID:AB_2057371), neuronal cell bodies (NeuN, Millipore, Darmstadt, Germany; Cat# MAB377, RRID:AB_2298772), non-phosphorylated neurofilaments indicative of axonal stress (SMI-32, Biolegend, San Diego CA, USA; Cat# 801701, RRID:AB_2564642) and accumulation of amyloid precursor protein (APP) indicative of axonal damage (1:2500, Millipore; Cat# MAB348, RRID:AB_94882). Following application of primary antibodies (except SMI-32), appropriate biotinylated secondary antibodies (Vector Labs) were applied, followed by reaction with ABC kit (Vector Labs) according to the manufacturer’s instructions (or in the case of APP, with avidin-coupled peroxidase, Sigma-Aldrich). Sections were then developed by incubating in 3,3′-diaminobenzidine (DAB, Sigma-Aldrich). For FoxP3-CD3 double labelling, CD3 was visualized by development with Vector SG as a substrate (Vector Labs) according to the manufacturer’s instruction allowing for combination with DAB-reacted FoxP3 immunolabelling. Following SMI-32 antibody application, fluorescent imaging was performed by incubation in a Cy3-conjugated secondary antibody (1:400, Jackson ImmunoResearch Labs Ely UK; Cat# 115-165-003, RRID:AB_2338680) and counterstaining with 4′,6-diamidino-2-phenylindole.

### Histopathological analyses

For all histopathological and immunohistochemical investigations, a minimum of 10 sections were taken throughout the length of the spinal cord. CC1, CD3, Mac-3 and APP immunohistochemistry was quantified as the number of positive cells or axons per mm^2^ of the total spinal cord (grey and white matter), whereas NeuN was quantified as positive cells per mm^2^ spinal cord grey matter. LFB staining was assessed semi-quantitatively in order to evaluate the extent of demyelination, using a scoring system as previously described [11, 12]. SMI-32 reactive density was quantified from images of both the posterior and anterior funiculi. Following background subtraction, pre-analytic equalizing of backgrounds was performed to account for variations in staining intensity. Using Image J analysis software (NIH, USA), images were adjusted to a monochrome 8-bit and a defined intensity threshold was set (35-255). SMI-32 positive axons were quantified using the analyse particles function with no size exclusion. Positive staining is given as percentage of white matter area. Microscopy was performed on an Eclipse 80i upright microscope (Nikon GmbH, Düsseldorf, Germany) with 2 ×, 10 ×, 20 × or 40 × objectives and fitted with a DXM1200C camera (Nikon). Although analyses were performed throughout the entire length of the spinal cord, all representative images shown are from the thoracic region, in order to aid comparison.

### Flow cytometry

Spleens were isolated on day 20 of EAE and mechanically dissociated using a 70-μm cell strainer, washed in ice cold PBS and erythrolysis performed using ACK lysing buffer (Thermo Fisher Scientific). T cells were then further isolated using a Pan T cell Isolation Kit according to the manufacturer’s instructions (Miltenyi Biotec GmbH, Bergisch Gladbach, Germany).

Following isolation, T cells were stimulated with 1 µg/ml ionomycin (Sigma-Aldrich), 5 µg/ml Brefeldin A (Sigma-Aldrich) and 20 ng/ml phorbol 12-myristate 13-acetate (Sigma-Aldrich) in RPMI-1640 medium (PAN Biotech) supplemented with 2 mM l-glutamine (Gibco), 10% FBS (Gibco), 100 U/ml penicillin, 0.1 mg/ml streptomycin (Sigma-Aldrich), 25 mM HEPES pH 7.4, 1 mM sodium pyruvate (PAA, Cambridge, UK), 0.1 mM non-essential amino acids solution (Lonza, Slough Wokingham, UK), 5 × 10^–5^ M 2-mercaptoethanol (Sigma-Aldrich) at a density of 0.1 × 10^6^ cells/ml for 5 h prior to staining. Cells were stained with the following antibodies against surface antigens: PE anti-mouse CD3 (BioLegend; Cat# 100206, RRID:AB_312663), FITC anti-mouse CD4 (BioLegend; Cat# 100406, RRID:AB_312691), PerCP anti-mouse CD8a (BioLegend; Cat# 100732, RRID:AB_893423). For intracellular staining, cells were fixed and permeabilized using Cytofix/Cytoperm^®^ (BD Biosciences) according to the manufacturer's protocol, followed by incubation with fluorescently labelled antibodies against intracellular molecules for 30 min at 4 °C: APC anti-mouse FoxP3 (eBioscience; Cat #FJK-16s, RRID: AB_469457), APC anti-mouse IL-17a (Thermo Fisher Scientific; Cat# 17-7177-81, RRID:AB_763580) and APC anti-mouse IFNγ (BioLegend; Cat# 505810, RRID:AB_315404).

Flow cytometry was performed using a FACSCanto II (BD Biosciences) with BD FACSDiva software, and analysed with Flow Jo software. The gating strategy was performed as is shown in Additional file 1: Fig. S1.

### Quantification of retinal ganglion cell density

Retinas were dissected, flat-mounted onto nitrocellulose membranes (Membrane Filter Black, white grid; GE Healthcare Life Sciences, Whatman TM, Chicago, IL, USA) with the ganglion cell layer upwards and fixed in 4% PFA. Immunolabelling for retinal ganglion cells (RGCs) was achieved using an antibody against RNA-binding protein with multiple splicing (RBPMS, 1:500, Abcam, Cambridge, UK; Cat #ab194213; RRID:AB_2920590).

To determine RGC cell densities, images were taken from all four retinal quadrants at three different locations (central, middle and peripheral positions corresponding to 1/6, 1/2 and 5/6 of the retinal radius), in order to adjust for local variance in RGC densities. Quantification was then performed manually from the images in an observer-blinded manner using an Image J cell counting plug-in (https://imagej.nih.gov/ij/).

### Western blotting

Retinal lysates were prepared by mechanical homogenization with ice‐cold lysis buffer (50 mM Tris HCl, 150 mM NaCl and 1% Triton X‐100) containing Complete Protease Inhibitor Cocktail (Roche Diagnostics GmBH, Mannheim, Germany), and sonicated for 5 s before clarification by centrifugation. A total of 30 µg protein was loaded onto a 4–20% gradient Mini‐PROTEAN^®^ TGX Stain‐Free™ Precast gel (BioRad, Hercules, CA, USA) and separated by SDS‐PAGE. Proteins were subsequently transferred to a polyvinylidene difluoride membrane for labelling with appropriate antibodies.

For detection of pAkt Ser473 (1:1000, Cell Signalling, Danvers, MA, USA; Cat #9271, RRID:AB_329825), Akt (1:1000, Cell Signalling; Cat #9272, RRID:AB_329827), pNF-κB p65 Ser536 (1:200, Cell Signalling; Cat# 3031, RRID:AB_330559) and NF-κB p65 (1:1000, Cell Signalling; Cat# 8242, RRID:AB_10859369), blocking was performed in 5% bovine serum albumin and 0.1% Tween 20 in Tris-buffered saline, whereas for glyceraldehyde 3‐phosphate dehydrogenase (GAPDH, 1:2000; Millipore; Cat# MAB374, RRID: AB_2107445) detection, blocking was performed in 5% milk powder and 0.1% Tween 20 in Tris-buffered saline. Membranes were incubated in primary antibodies overnight at 4 °C after which visualization was performed using appropriate horse radish peroxidase (HRP)‐conjugated secondary antibodies (donkey anti-rabbit HRP-linked, 1:5000, Cytiva, Shrewsbury, MA, USA; Cat# GENA934, RRID:AB_2722659 or sheep anti-mouse HRP-linked, 1:5000, Cytiva; Cat# NA931, RRID:AB_772210), followed by ECL Prime reagent (Amersham, Bucks, UK) and imaged using a ChemiDoc XRS + Imaging System (BioRad).

### Statistics

Data were analysed using GraphPad Prism 8 (GraphPad Software, La Jolla, CA, USA), and are presented as mean ± standard error of the mean (SEM). Data sets were tested for normality using the Shapiro–Wilk test. Subsequently a one-way ANOVA with post hoc Dunnett’s test (for parametric data) or Dunn’s test (for non-parametric data) was performed. A *p* value < 0.05 was considered to be statistically significant.

## Results

### TNFR modulation ameliorates EAE

To determine whether the strategy of antagonizing TNFR1 simultaneously with TNFR2 agonism was effective under neuroinflammatory conditions, an animal model of MS, EAE, was induced in mice in which the extracellular part of human TNFR1 is fused to the trans-membrane and intracellular region of mouse TNFR1, termed hu/m TNFR1ki [15].

**Fig. 1:**
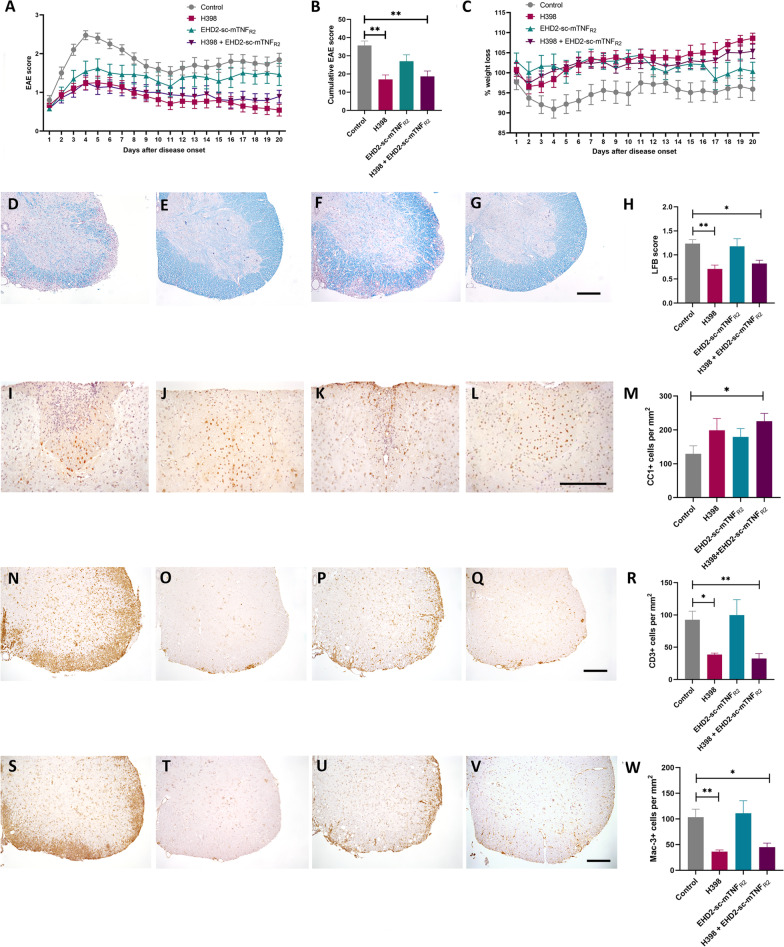
TNFR modulation ameliorates EAE, reducing demyelination, oligodendrocyte loss and cellular infiltration. hu/m TNFR1ki mice were treated with either PBS (control) (*n* = 20), 20 mg/kg H398 (*n* = 24), 10 mg/kg EHD2-sc-mTNF_R2_ (*n* = 13) or H398 and EHD2-SC-mTNF_R2_ (*n* = 24) on days 1, 4, 8, 12 and 16 of manifest EAE and followed until day 20 of EAE (**A**). Cumulative EAE scores (**B**) and evaluation of EAE-associated weight loss (**C**) were then assessed. hu/m TNFR1ki mice treated with either PBS (**D**, **I**,** N, S**; *n* = 10), H398 (**E**, **J**, **O, T**; *n* = 10), EHD2-sc-mTNF_R2_ (**F**, **K**, **P, U**; *n* = 8) or H398 and EHD2-SC-mTNF_R2_ (**G**, **L**, **Q, V**; *n* = 9) were killed at day 20 of EAE. Spinal cord sections were assessed for either **H** demyelination using LFB, **M** the density of CC1-positive oligodendrocytes, **R** CD3-positive T cell infiltration, or **W** the density of Mac-3-positive macrophages/activated microglia. *p < 0.05, **p < 0.01, cumulative score and LFB one-way ANOVA followed by Dunn’s multiple comparison test, CC1, CD3 and Mac-3 one-way ANOVA followed by Dunnett’s multiple comparison test. Scale bars = 100 µm

Following immunization, mice were treated from the first day of manifest EAE when spinal cord symptoms become apparent (EAE day 1) with either 20 mg/kg of the TNFR1-specific antagonistic antibody H398, 10 mg/kg of the TNFR2-specific agonist EHD-sc-mTNF_R2_, a combination of the two, or with PBS as a control. Treatment was repeated on days 4, 8, 12 and 16 of EAE and animals killed on day 20 of EAE. As we have previously shown [11, 12], treatment with an anti-TNFR1 antibody was very effective in reducing the severity of EAE, as assessed by spinal cord motor symptoms, in comparison to PBS-treated mice (Fig. 1A,) resulting in a highly significant reduction in the cumulative EAE score (control 35.69 ± 2.45; H398, 16.96 ± 2.52, *p* = 0.0003; EHD-sc-mTNF_R2,_ 27 ± 3.55; H398 + EHD-sc-mTNF_R2,_ 18.67 ± 2.97, *p* = 0.0009; Fig. 1B). Furthermore, H398 also prevented EAE-associated weight loss, which is frequently used as an indicator of general well-being (Fig. 1C). It is notable however, that under the treatment conditions applied, the TNFR2 agonist EHD-sc-mTNF_R2_ alone had only a modest effect on disease severity and the combination of H398 and EHD-sc-mTNF_R2_, provided no additional benefit to inhibiting TNFR1 alone.

### TNFR modulation reduced demyelination, oligodendrocyte loss and inflammatory infiltration

The major pathological hallmark of EAE is inflammatory demyelination. To assess the efficacy of TNFR modulation to reduce demyelination, we performed LFB staining of spinal cord sections from animals at day 20 of EAE. Consistent with the reduction in the severity of EAE symptoms, mice treated with H398 and those treated with H398 together with EHD-sc-mTNF_R2_, had significantly less spinal cord demyelination than control-treated animals. Mice treated with EHD-sc-mTNF_R2_ alone, however, had a similar level of demyelination as mice in the control group (control, 1.24 ± 0.08; H398, 0.71 ± 0.08, *p* = 0.002; EHD-sc-mTNF_R2,_ 1.18 ± 0.16; H398 + EHD-sc-mTNF_R2,_ 0.82 ± 0.07, *p* = 0.0348; Fig. 1D–H). Correspondingly, following TNFR modulation, there was an increase in the number of CC1-positive differentiated/myelinating oligodendrocytes. However, this reached significance in comparison to controls only in the group treated with both H398 and EHD-sc-mTNF_R2_ (control, 129.44 ± 13.94; H398, 199.00 ± 4.74; EHD-sc-mTNF_R2,_ 179.35 ± 17.92; H398 + EHD-sc-mTNF_R2,_ 225.94 ± 016.73, *p* = 0.0367; Fig. 1I–M).

In order to determine whether TNFR modulation also affected inflammatory infiltration we performed immunohistochemistry, firstly with an antibody against CD3 to detect T cells (Fig. 1N–R), and secondly with the antibody Mac-3 which detects activated microglia and macrophages (Fig. 1S–W). Inhibition of TNFR1 with H398 caused a significant reduction in the infiltration of both T cells and activated microglia/macrophages, as did treatment with a combination of H398 and EHD-sc-mTNF_R2_. Agonism of TNFR2 with EHD-sc-mTNF_R2_ alone did not, however, affect inflammatory infiltration of either T cells or macrophages/microglia (CD3, control 92.6 ± 13.19; H398, 38.7 ± 2.18, *p* = 0.0131; EHD-sc-mTNF_R2,_ 99.75 ± 23.91; H398 + EHD-sc-mTNF_R2,_ 32.56 ± 7.58, *p* = 0.0068. Mac-3, control 103.2 ± 15.93; H398, 36.3 ± 3.2, *p* = 0.0041; EHD-sc-mTNF_R2,_ 111.1 ± 24.27; H398 + EHD-sc-mTNF_R2,_ 45 ± 7.846, *p* = 0.0163).

### TNFR modulation does not alter immune cell phenotype in chronic EAE

**Fig. 2:**
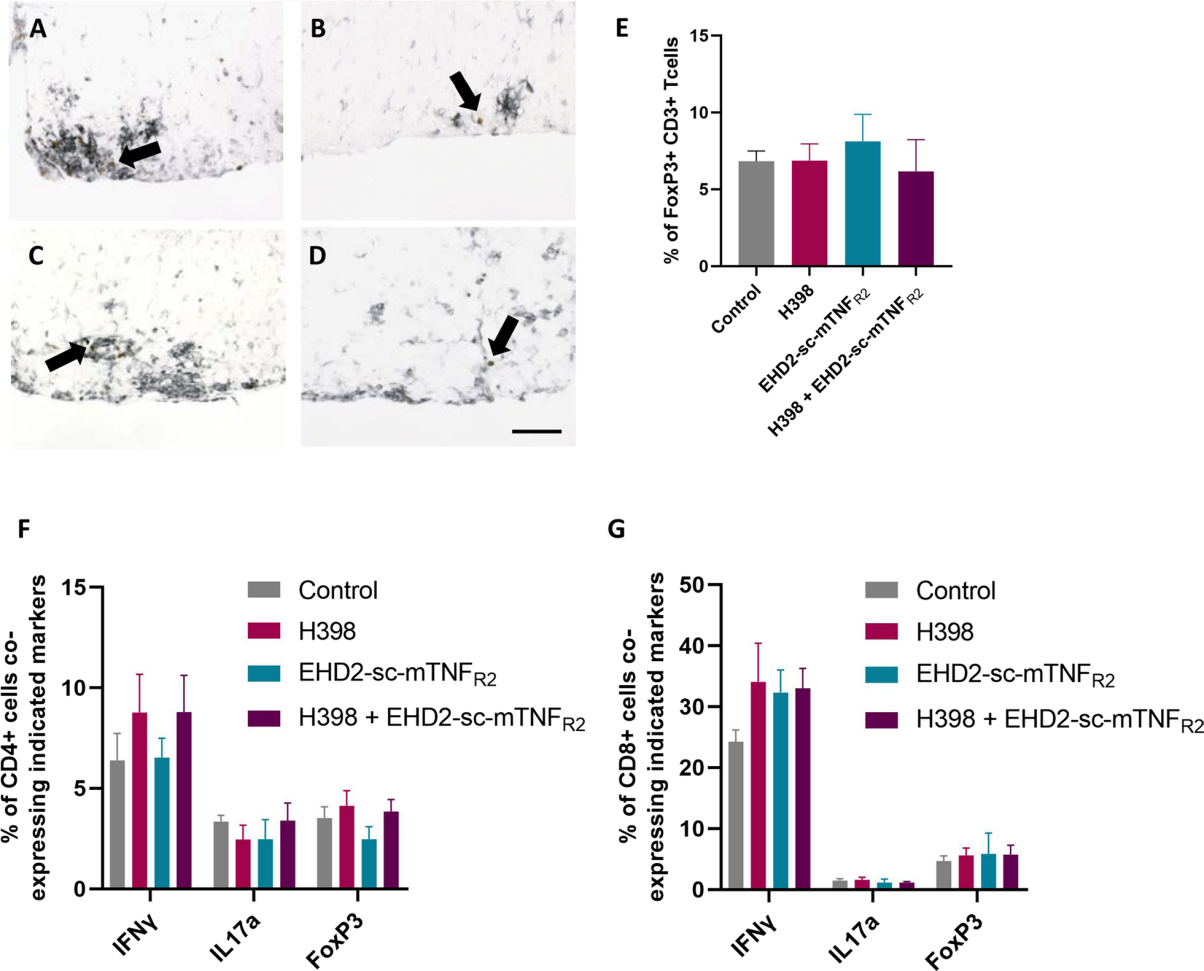
TNFR modulation does not affect immune cell phenotype. Hu/m TNFR1ki mice treated with either PBS (control) (**A**; *n* = 6), H398 (**B**; *n* = 8), EHD2-sc-mTNF_R2_ (**C**; *n* = 5), or H398 and EHD2-SC-mTNF_R2_ (**D**; *n* = 5) and were killed at day 20 of EAE. Spinal cord sections were immunolabelled with antibodies against both CD3 and FoxP3 to detect regulatory T cells using Vector SG (black) as a chromogen to detect CD3-positive cells and DAB (brown) to detect FoxP3-positive cells. **E** Quantification of double-labelled CD3/FoxP3-positive cells. For flow cytometry, splenocytes were isolated from mice treated with either H398 (*n* = 7), EHD2-sc-mTNF_R2_ (*n* = 4), H398 and EHD2-SC-mTNF_R2_ (*n* = 8) or PBS (*n* = 6) on day 20 of EAE. The percentage of **F** CD4-positive or **G** CD8-positive T cells co-expressing markers of either TH1 (IFNγ), TH17 (IL-17a), or Tregs (FoxP3) was assessed. Scale bar = 50 µm

One reason that targeting TNFR2 has been postulated as a potential therapeutic strategy is due to its role in the promotion of regulatory T cell activity and expansion [22]. We therefore wished to investigate whether the modulation of TNFR activity would lead to an alteration in either the peripheral or central nervous system (CNS) immune cell phenotype. We performed immunohistochemistry using a combination of antibodies against both CD3 and FoxP3 on spinal cord sections from day 20 of EAE. However, we did not see any change in the proportion of CD3-positive T cells co-expressing FoxP3 in any of the treatment groups (control, 6.83 ± 0.66; H398, 6.87 ± 1.08; EHD-sc-mTNF_R2,_ 8.12 ± 1.76; H398 + EHD-sc-mTNF_R2,_ 6.17 ± 2.06; Fig. 2A–E). To investigate the peripheral immune cell phenotype, splenocytes were obtained at day 20 of EAE and CD3-positive T cells were isolated. Using flow cytometry, T cells were gated for expression of either CD4 or CD8, and analysed to determine the relative proportion of these subpopulations co-expressing markers of TH1 (IFNγ-positive), TH17 (IL-17a-positive), and Treg (FoxP3-positive) subsets. Again, we did not see any alteration in the proportions of either CD4- or CD8-positive cells expressing these markers under any of the treatment conditions (Fig. 2F, G).

### TNFR modulation protects axons during EAE

**Fig. 3:**
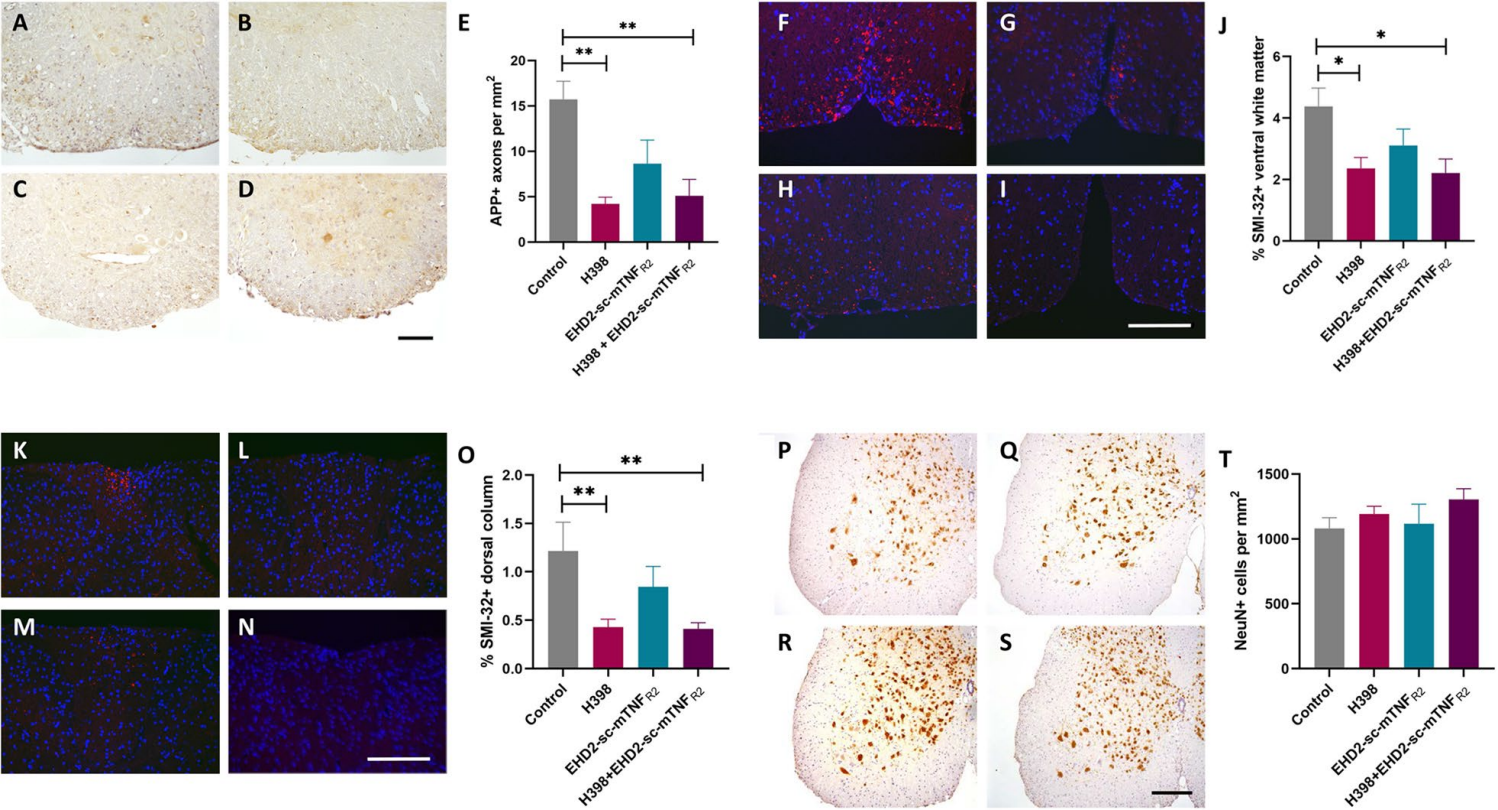
TNFR modulation protects against axonal degeneration in EAE. Hu/m TNFR1ki mice treated with either H398 (**B**, **G**, **L, Q**; *n* = 10), EHD2-sc-mTNF_R2_ (**C**, **H**, **M, R**; *n* = 5), H398 and EHD2-sc-mTNF_R2_ (**D**,** I**, **N, S**; *n* = 9) or PBS (**A**, **F**,** K, P**; *n* = 10) were killed at day 20 of EAE. **A–D** Axonal damage within the spinal cord was assessed by immunohistochemistry using an antibody against APP, and quantified (**E**). The presence of non-phosphorylated neurofilament-H (a marker of axonal stress) was assessed within the spinal cord using the SMI-32 antibody, in both **F–I** ventral and **K–N** dorsal regions. The area of SMI-32 positivity was then quantified in both the **J** ventral and **O** dorsal white matter. **P–S** The density of motor neuronal cell bodies within the spinal cord grey matter was assessed using an antibody against NeuN, with quantification (**T**). *p < 0.05, **p < 0.01, one-way ANOVA followed by Dunn’s multiple comparison test. Scale bars = 100 µm

Axonal degeneration is thought to be the major pathological correlate of disability in both MS and EAE [23, 24]. To determine if TNFR modulation reduced axonal damage we performed immunohistochemistry with an antibody against the fast-transported amyloid precursor protein (APP), to detect axonal transport deficits indicative of axonal damage. Antagonism of TNFR1 with H398 led to a significant reduction in APP-positive axons (control, 15.73 ± 1.99; H398, 4.21 ± 0.73; *p* = 0.0058), as did the combination treatment with H398 and EHD-sc-mTNF_R2_ (5.10 ± 1.81, *p* = 0.0063), compared to control-treated mice. However, the apparent reduction in the number of damaged axons following treatment with EHD-sc-mTNF_R2_ alone was not significant (8.64 ± 2.60, *p* = 0.2633) (Fig. 3A–E).

These results were confirmed by immunohistochemistry with the antibody SMI-32 to detect non-phosphorylated neurofilament-H, in both the ventral (Fig. 3F–J) and dorsal (Fig. 3K–O) spinal cord of mice at day 20 of EAE. Similar to APP, H398 alone and combination of H398 with EHD-sc-mTNF_R2_ caused a significant reduction in axonal degeneration (ventral spinal cord, control 4.40 ± 0.60; H398, 2.64 ± 0.36, *p* = 0.0421; H398 + EHD-sc-mTNF_R2,+_ 2.21 ± 0.46, *p* = 0.0227; dorsal spinal cord, control 1.22 ± 0.30; H398, 0.4287 ± 0.08, *p* = 0.0081; H398 + EHD-sc-mTNF_R2,+_ 0.41 ± 0.06, *p* = 0.0051;). Again, an apparent reduction in SMI-32 immunoreactivity in mice treated with EHD-sc-mTNF_R2_ alone was not significant (ventral spinal cord, EHD-sc-mTNF_R2,_ 3.11 ± 0.54, *p* = 0.5010; dorsal spinal cord, EHD-sc-mTNF_R2,_ 0.85 ± 0.21, *p* = 0.8978).

### Antagonism of TNFR1 concomitant with agonism of TNFR2 is neuroprotective in EAE

To determine whether TNFR modulation could be neuroprotective, we firstly assessed the number of NeuN-positive neurons within the spinal cord grey matter. Although treatment with a combination of H398 and EHD-sc-mTNF_R2_ led to an increase in the number of NeuN-positive neuronal cell bodies in comparison to control-treated mice, this was not significant (H398 + EHD-sc-mTNF_R2_, 1304.14 ± 83.31; control, 1080.74 ± 82.61, *p* = 0.233) (Fig. 3P–T). No change in the survival of neuronal cell bodies could be seen in any of the other treatment groups.

**Fig. 4:**
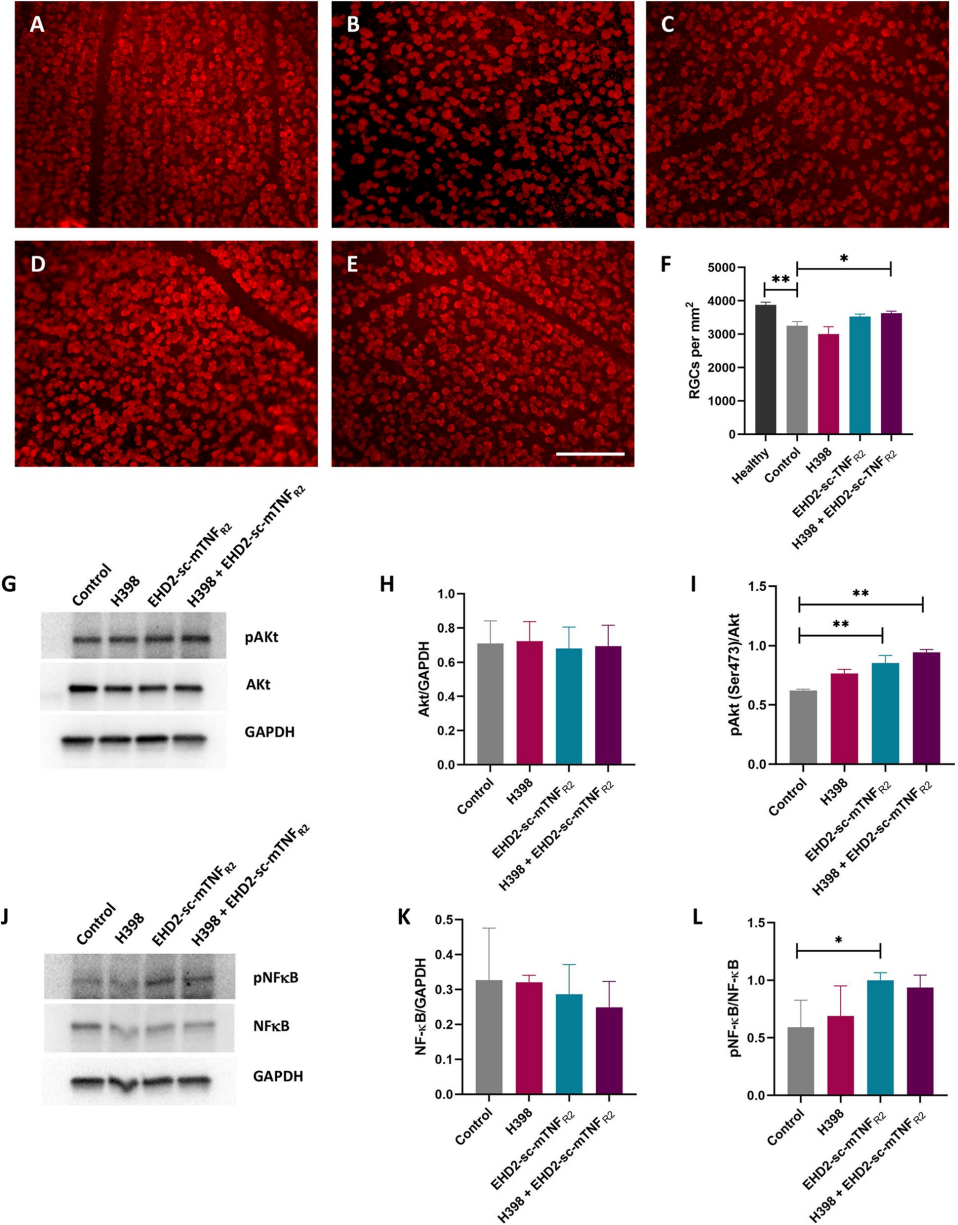
TNFR modulation is neuroprotective and promotes neuroprotective signalling pathways. Whole-mounted retinas were immunolabelled with an antibody against RBPMS to identify surviving RGCs for quantification. Representative images are shown from healthy hu/m TNFR1ki mice (**A**), or EAE mice treated with either PBS (control, **B**), H398 (**C**), EHD2-sc-mTNF_R2_ (**D**), or a combination of H398 and EHD2-sc-mTNF_R2_ (**E**). EAE retinas were extracted on day 20 of EAE and quantification performed (**F**; healthy, *n* = 8 retinas from 4 mice; control, *n* = 13 retinas from 7 mice; H398, *n* = 14 retinas from 8 mice; EHD2-sc-mTNF_R2_, *n* = 8 retinas from 5 mice; H398 and EHD2-sc-mTNF_R2_, *n* = 13 retinas from 7 mice). Western blotting was performed to determine the protein levels of **G** phosphorylated Akt (pAkt (ser 473), 62 kDa), Akt (62 kDa) and GAPDH (37 kDa), or **J** for phosphorylated NF-κB p65 subunit (pNF-κB (ser536), 65 kDa), NF-κB (65 kDa), and GAPDH (37 kDa) on unfractionated retinal lysates prepared on day 20 of EAE. Quantification (*n* = 4 per treatment group) was performed to assess the ratios of **H** Akt/GAPDH, **I** pAkt/Akt, **K** NF-κB/GAPDH, or **L** pNF-κB/NF-κB. Uncropped Western blots are shown in Additional file 2: Fig. S2. *p < 0.05, **p < 0.01, one-way ANOVA followed by Dunn’s multiple comparison test (**F**), one-way ANOVA followed by Dunnett’s multiple comparison test (**H**, **I**, **K**, **L**). Scale bar = 100 µm

Next, we studied the survival of RGCs, a discrete population of neurons affected in EAE [25] using flat-mounted retinas which were immunolabelled to identify cells positive for the RGC-specific marker RBPMS [26]. At day 20 of EAE, in the control group there was an approximate 20% loss of RGCs in comparison to healthy retinas (healthy, 3878 ± 82.7; control-treated, 3253.29 ± 118.35 RBPMS-positive RGCs per mm^2^, *p* = 0.0009). Following treatment with a combination of treatment with H398 and EHD-sc-mTNF_R2_, there were significantly more surviving RGCs (H398 + EHD-sc-mTNF_R2_, 3626.81 ± 65.76; control, 3253.29 ± 118.35, *p* = 0.0467) (Fig. 4A–F). Treatment with EHD-sc-mTNF_R2_ alone led to an increase, though non-significant, in surviving RGCs compared to healthy retinas (3532.29 ± 64.71, *p* = 0.1754), whereas treatment with H398 alone saw no increase in RGC survival (3003 ± 195.18, *p* > 0.99).

### Modulation of TNFR1 and TNFR2 promotes the upregulation of neuroprotective signalling pathways

To understand the underlying mechanisms that mediate the protection of RGCs that we observed following treatment with H398 and EHD-sc-mTNF_R2_, unfractionated retinal lysates were prepared from mice at day 20 of EAE and Western blotting was performed. Since it has previously been shown to be involved in TNFR2-mediated neuroprotection, we initially investigated the PI3K–Akt pathway [3]. Since phosphorylation at serine-473 leads to the activation of Akt, we investigated the ratio of pAkt/Akt. Whilst the overall level of Akt expression in comparison to GAPDH did not increase in any of the treatment groups, there was a significant increase in the ratio of pAkt/Akt, in both the EHD-sc-mTNF_R2_ group and the H398 and EHD-sc-mTNF_R2_ treated group (control 0.62 ± 0.01; H398 0.77 ± 0.03; EHD-sc-mTNF_R2,_ 0.85 ± 0.06, *p* = 0.0073; H398 + EHD-sc-mTNF_R2_ 0.94 ± 0.02, *p* = 0.001) (Fig. 4G–I). Furthermore, as TNFR2 is also known to promote neuroprotection via phosphorylation and thus activation of NF-κB [27], we performed Western blotting with antibodies against the phosphorylated NF-κB p65 subunit and NF-κB. Again, there was no increase in the overall level of NF-κB expression in comparison to GAPDH in any treatment group. Following treatment with H398 alone, there was no change in the ratio of pNF-κB/NF-κB, whereas with EHD-sc-mTNF_R2_ alone, there was a significant increase in the ratio of pNF-κB/NF-κB. However, the increase in the ratio of pNF-κB/ NF-κB in the combination treatment group did not quite reach significance (control 0.59 ± 0.12; H398, 0.69 ± 0.13; EHD-sc-mTNF_R2_ 1.00 ± 0.03, *p* = 0.0245; H398 + EHD-sc-mTNF_R2,_ 0.94 ± 0.057) (Fig. 4J–L).

## Discussion

MS is an autoimmune disease in which neurodegeneration is the underlying cause of permanent disability [23] and yet until now, all approved therapies are only targeted towards the inflammatory aspect of the disease. Modulation of TNF activity, a master regulator of both inflammation and, as has been revealed more recently, neuronal survival, may allow both disease aspects to be modified. TNF is thought to play a critical role in the pathogenesis of MS; in the normal adult brain TNF is expressed at low levels [28], however in post-mortem studies, increased levels of TNF were observed in MS brains associated with lesions [29, 30]. Furthermore, levels of TNF in the sera and CSF of MS patients have been found to correlate with disease activity and progression [2, 31–33].

The potential role played by TNF in the pathogenesis of MS was further highlighted in a large genome-wide association study, where a single nucleotide polymorphism associated with increased risk of developing MS was found in the *TNFRSF1A* gene that encodes TNFR1 [34]. It was subsequently reported that this polymorphism led to the expression of a novel form of sTNFR1 that antagonizes TNF [35], a finding consistent with the detrimental effects of anti-TNF therapy in patients [5].

Further evidence that sTNFR1 may be involved in the disease pathogenesis of MS comes from a study in which a positive association between plasma levels of sTNFR1 and both disability and disease progression was observed, whereas conversely, there was a negative association between sTNFR2 levels and the development of progressive forms of MS [36]. A study of grey matter pathology has additionally indicated that an imbalance of TNFR1/TNFR2 signalling may play a role in determining the severity of MS. Analysis of post-mortem MS cortices revealed a positive correlation between meningeal inflammation and the expression of genes involved in TNFR1 signalling, and conversely, areas of reduced inflammation had greater expression of genes associated with TNFR2 signalling [37].

Due to the dual function of TNFR1 and TNFR2, several studies have successfully targeted the pro-inflammatory TNFR1 signalling in EAE using different pharmacological approaches, whilst leaving TNFR2 signalling untouched. Amongst others, strategies have included inhibiting soluble TNF [10], using a TNF mutein PEGylated R1antTNF [13], as well as human TNFR1-specific antagonistic antibodies [11, 12, 38].

The alternative approach of promoting protective TNFR2 signalling has also been investigated in various disease models through the use of a growing number of agonists. For example, the TNFR2-specific agonist TNCscTNF80, was protective in a model of autoimmune arthritis [39]. Similarly, TNFR2 agonism with STAR2 was also beneficial in a model of graft-versus-host disease [40], whereas NEWSTAR2 was recently shown to ameliorate neuropathology in a model of Alzheimer’s disease [41]. The human TNFR2 agonist EHD2-sc-TNF_R2_ has previously been shown to protect primary cortical neurons from glutamate toxicity in vitro as well as to have neuroprotective effects in vivo using the nucleus basalis lesion model [15]. The murine orthologue of this compound, EHD-sc-mTNF_R2_, which was used in this study, also alleviated disease in models of collagen-induced arthritis [17], neuropathic pain [42] as well as traumatic spinal cord injury [43].

Positive effects of TNFR2 modulation have also been reported already in EAE, which may arise from several different mechanisms. EHD2-sc-mTNF_R2_, the agonist used in this study, was used successfully to treat EAE symptoms [44], with its positive effects attributed in part to the promotion of peripheral Treg expansion and subsequent suppression of autoimmunity. This agrees with the described role of TNFR2 signalling in Treg expansion, as well as in regulating Treg responses to TCR stimulation and their suppressive function [45]. Similarly, the specific deletion of TNFR2 on Tregs led to an increase in EAE disease severity [46], demonstrating its role in suppressing autoimmune disease. However in this study, we did not see a significant effect of the TNFR2 agonist on the disease course of EAE in the absence of TNFR1 antagonism, and also no significant effects on either CNS or peripheral Treg populations. It is not clear why this result differs from previously published studies, but may reflect the dominant effect of TNFR1 in this disease model.

An alternative mechanism for the protective effects of TNFR2 agonism in EAE may be through the protection of oligodendrocyte lineage cells from oxidative stress-mediated cell death [47]. This was hinted at by our observation of significant protection of CC1-positive oligodendrocytes with a combinatorial treatment approach, although this may also reflect the reduced pro-inflammatory environment following TNFR modulation. In addition, TNFR2 agonism might also influence both oligodendrocyte lineage cell differentiation [48] and, as has been described recently, their immune-modulatory capacity [49, 50]. Furthermore, the function of TNFR2 in promoting remyelination has been demonstrated in both EAE and following cuprizone-mediated demyelination, in both global TNFR2−/− mice [51] and mice lacking TNFR2 specifically in oligodendrocytes [48]. However, the study performed here was not designed to address remyelination since for significant remyelination to occur, a longer timeframe has been reported to be necessary [10, 52].

Due to the dual, but opposing, effects of TNFR1 and TNFR2 that have been reported, it has been suggested that the simultaneous inhibition of TNFR1 whilst promoting TNFR2 signalling might be potentially beneficial in the treatment of MS [4]. This approach was also suggested following observations that the protective effects of TNFR2 agonism with EHD2-scTNF_R2_ on primary cortical neurons treated with NMDA could be enhanced by TNFR1 inhibition; and conversely, the protective effects of TNFR1 inhibition using the antagonistic antibody ATROSAB following injection of NMDA into the nucleus basalis magnocellularis could be abolished by TNFR2 inhibition [15]. Thus, the protective effects of both EHD2-scTNF_R2_ and ATROSAB appear to require simultaneous suppression of TNFR1 and activation of TNFR2 signalling, and co-modulation may be most effective.

This was the therapeutic strategy addressed in the current study and, in agreement with this hypothesis, we saw a significant neuroprotection of RGCs only when both TNFRs were targeted, although this was not seen in the spinal cord, possibly reflecting the robust neurodegeneration observed in the retina in MOG-induced EAE [25]. Although both TNFR1 and 2 targeting was necessary for significant protection of RGCs, increases in both Akt and NF-κB phosphorylation within the retina following EHD2-sc-mTNF_R2_ treatment was observed whether given alone or in combination with H398. It has been previously reported that EHD2-scTNF_R2_ may exert direct neuroprotective effects through a PI3K-PKB/Akt-mediated pathway [14, 53, 54], and that TNFR2 can activate both canonical and non-canonical NF-κB pathways [53, 55, 56]. Thus, the combinatorial approach may take advantage of protecting against neuroinflammation (TNFR1) and promoting cell survival signalling (TNFR2).

Collectively, we demonstrate, using the EAE model of autoimmunity, that the novel combined approach of concomitantly inhibiting TNFR1 whilst promoting TNFR2 signalling may achieve greater beneficial effects than either approach in isolation. This in turn may explain some of the previous failings in modulating TNF activity in autoimmune disease, as well as strengthening the evidence that this novel combinatorial approach may serve as a new direction in TNF-targeting therapies.

## Supplementary Information

**Additional file 1: Fig. S1**. Flow cytometry gating strategy.

**Additional file 2: Fig. S2**. Full uncropped Western blot images.

## Data Availability

The datasets used and/or analysed during the current study are available from the corresponding author on reasonable request.

## Abbreviations

APP: Amyloid precursor protein
CNS: Central nervous system
DAB: 3,3′-Diaminobenzidine
EAE: Experimental autoimmune encephalomyelitis
GAPDH: Glyceraldehyde 3-phosphate
HRP: Horse radish peroxidase
IFNγ: Interferon gamma
IL-17a: Interleukin 17a
LFB: Luxol fast blue
MOG: Myelin oligodendrocyte glycoprotein
MS: Multiple sclerosis
NMDA: *N*-Methyl-d-aspartate
PBS: Phosphate-buffered saline
PFA: Paraformaldehyde
RBPMS: RNA-binding protein with multiple splicing
RGC: Retinal ganglion cell
SEM: Standard error of the mean
sTNF: Soluble tumour necrosis factor
TACE: TNF-α-converting enzyme
tmTNF: Transmembrane tumour necrosis factor
TNF: Tumour necrosis factor α
TNFR1: Tumour necrosis factor receptor-1
TNFR2: Tumour necrosis factor receptor-2
