# Supplementary material for: Co-modulation of TNFR1 and TNFR2 in an animal model of multiple sclerosis
Source: J Neuroinflammation. 2023 Apr 30;20:100. doi: 10.1186/s12974-023-02784-z (PMC10149004; doi:10.1186/s12974-023-02784-z)
Supplement: Supplementary file 1 — Additional file 1: Fig. S1. Flow cytometry gating strategy. [file 12974_2023_2784_MOESM1_ESM.pdf]

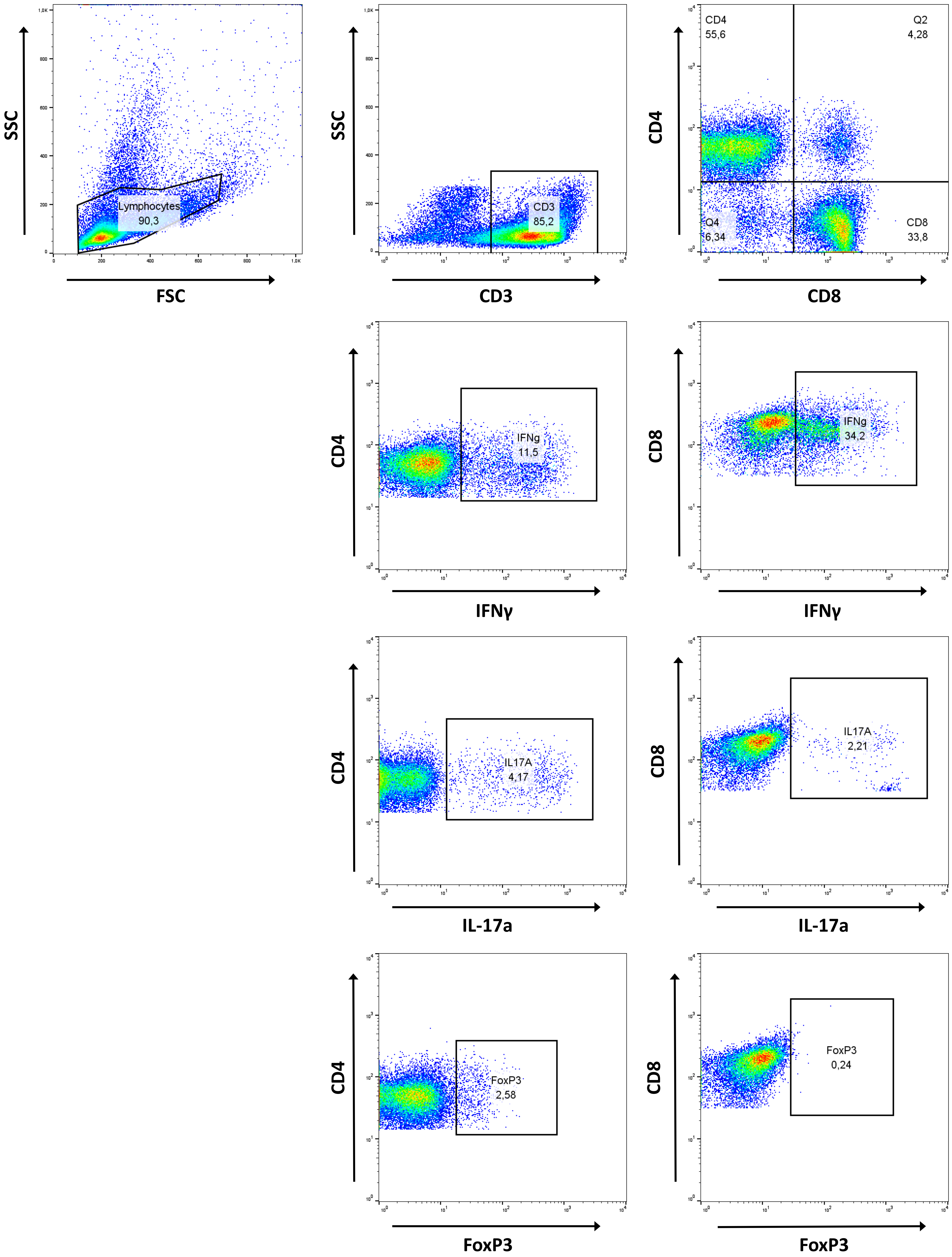

SSC
FSC
Lymphocytes
90,3
SSC
CD3
CD3
85,2
CD4
CD8
CD4
55,6
Q2
4,28
Q4
6,34
CD8
33,8
CD4
IFNγ
IFNg
11,5
CD8
IFNγ
IFNg
34,2
CD4
IL-17a
IL17A
4,17
CD8
IL-17a
IL17A
2,21
CD4
FoxP3
FoxP3
2,58
CD8
FoxP3
FoxP3
0,24

Fig. S1. Flow cytometry gating strategy. T cells were isolated and immunolabelled as indicated in the Methods. Representative dot plots (generated with FlowJo) are shown for the gating performed for data shown in Figure panels 2F and 2G. Cells were gated for lymphocytes based on forward (FSC) and side (SSC) scattering, before further gating for expression of CD3 and either CD4 or CD8. CD4+ and CD8+ T cells were subsequently assessed for the percentage co-expressing either IFNγ (TH1 T cells), IL-17a (TH17 T cells) or FoxP3 (Tregs).
