# Supplementary figures and images for: Co-modulation of TNFR1 and TNFR2 in an animal model of multiple sclerosis
Source: J Neuroinflammation. 2023 Apr 30;20:100. doi: 10.1186/s12974-023-02784-z (PMC10149004; doi:10.1186/s12974-023-02784-z)

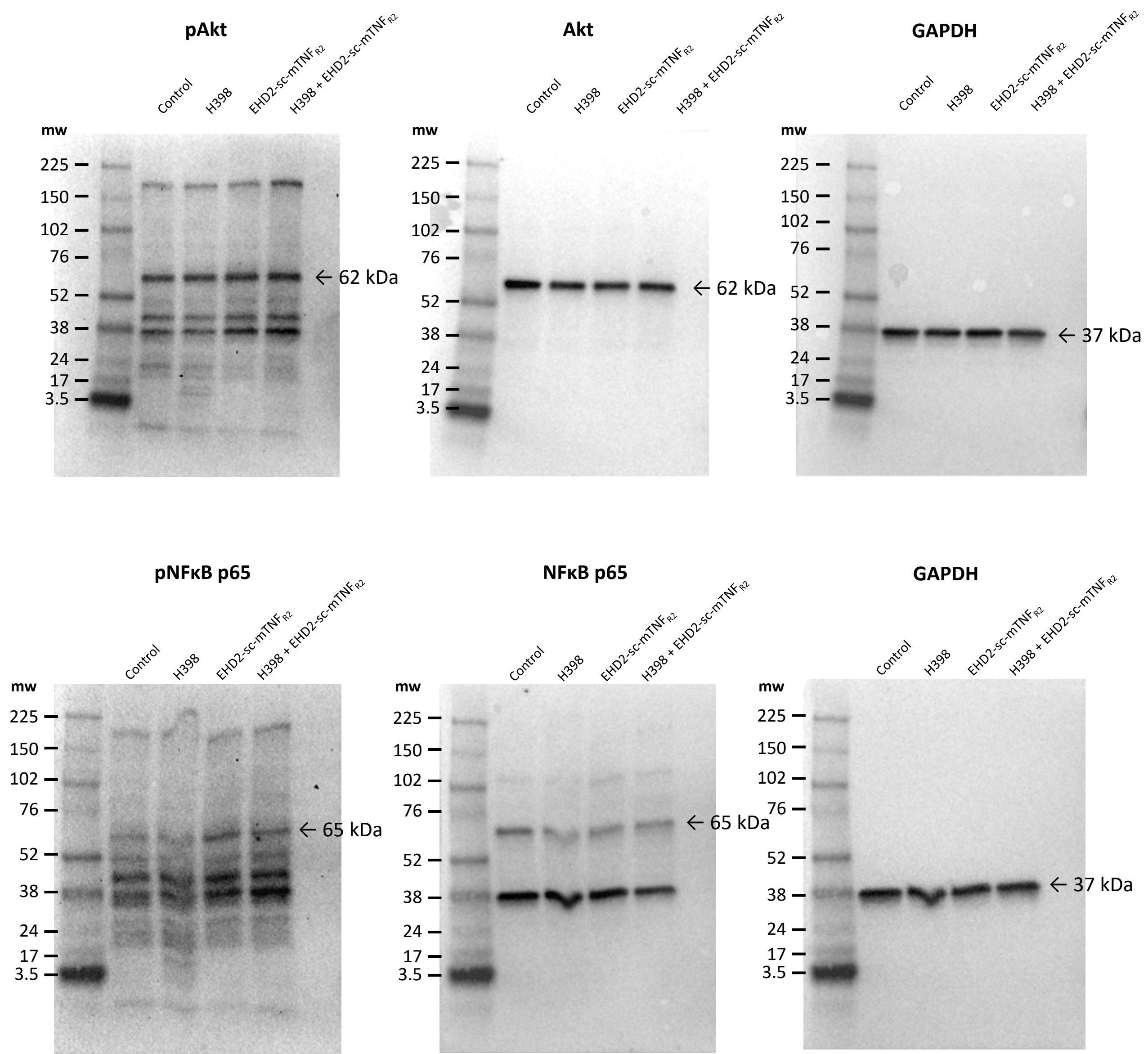

**Fig. S2.** Full uncropped Western blot images corresponding to (A) Figure 4G and (B) Figure 4J.

Supplement: Supplementary file 2 — Additional file 2: Fig. S2. Full uncropped Western blot images. [file 12974_2023_2784_MOESM2_ESM.pdf]
